# Surgery in the combination therapies era

**DOI:** 10.1186/1479-5876-13-S1-K11

**Published:** 2015-01-15

**Authors:** Nicola Mozzillo

**Affiliations:** 1 Director of the Department Melanoma and Soft Tissues, National Cancer Institute “Fondazione G. Pascale” of Naples, Italy

## 

Stage IV melanoma has historically had a poor prognosis because of lack of responsiveness to traditional chemotherapeutics. The 1-year survival rate of patients diagnosed with stage IV melanoma varies between 33 and 66 %. Depending on the number, the location, and the resectability of distant metastases, common treatment options comprise surgery, systemic medical therapy, and radiotherapy.

Single-agent DTIC has been the standard chemotherapy regimen for metastatic melanoma since 1970s. From a recent meta-analysis Luo et al [1] demonstrated that the substantial benefit from any of the various combinations of chemotherapy and biochemotherapy is about 4.2% with a mean overall survival of 6 months.

Patients who have limited sites of metastatic disease, a long disease-free survival and a tumor doubling time higher than 60 days, may be amenable to surgical resection and complete surgical excision of limited metastatic disease can result in prolonged relapse-free survival in carefully selected patients. The potential for complete surgical resection to benefit individual patients is suggested by the results of a prospective, phase II study from the Southwest Oncology Group. In this prospective, multicenter study, 64 of 77 carefully selected patients were able to undergo complete resection of all sites of metastatic disease. Overall, the three and four-year survival rates were 36 and 31 percent, respectively, although late relapses continued to be observed after this time.

Ipilimumab is a fully human monoclonal antibody directed against cytotoxic T-lymphocyteassociated antigen-4 (CTLA-4), a negative regulator of T-cell-mediated immune responses. In Phase III trials, ipilimumab treatment significantly extended overall survival (OS) compared with control in both pretreated and treatment-naϊve patients. Approximately 10% of patients present objective responses by standard criteria, whereas 10–20% have stable disease or minor responses, that translate into a clinical benefit. In patients who initially achieve clinical benefit and then relapse months or years later, re-treatment with ipilimumab can be also advantageous.

Vemurafenib stands as the first personalized treatment in melanoma based on a specific mutation, with a favorable impact on survival. The overall survival rate at 6 months was 77% and 58% at 12.

As a consequence, future improvements of this targeted and personalized approaches are expected from ongoing clinical trials aiming at potentiate the activity of BRAF inhibitors through combination with other molecules. These combination therapies also aim at lowering the observed skin toxicities.
